# Draft Genome Sequence of *Rhodoplanes* sp. Strain T2.26MG-98, Isolated from 492.6 Meters Deep on the Subsurface of the Iberian Pyrite Belt

**DOI:** 10.1128/MRA.00070-19

**Published:** 2019-04-18

**Authors:** N. Mariñán, José M. Martínez, T. Leandro, R. Amils

**Affiliations:** a Centro de Biología Molecular Severo Ochoa (CBMSO, CSIC-UAM), Universidad Autónoma de Madrid, Cantoblanco, Madrid, Spain; b Center for Neuroscience and Cell Biology, University of Coimbra, Coimbra, Portugal; c Centro de Astrobiología (CAB, CSIC-INTA), Torrejón de Ardoz, Madrid, Spain; University of Arizona

## Abstract

*Rhodoplanes* sp. strain T2.26MG-98 was isolated from the deep subsurface of the Iberian Pyrite Belt. We report its draft genome, consisting of 214 contigs with a chromosome of ∼5.6 Mb and a 53.7-kb plasmid. The chromosome annotation identified 4,994 coding DNA sequences, 1 rRNA operon, and 57 tRNA genes.

## Abstract (precis)

*Rhodoplanes* sp. strain T2.26MG-98 was isolated from the deep subsurface of the Iberian Pyrite Belt. We report its draft genome, consisting of 214 contigs with a chromosome of ∼5.6 Mb and a 53.7-kb plasmid.

## ANNOUNCEMENT

*Rhodoplanes* is a facultative anoxygenic phototroph genus classified within the family *Hyphomicrobiaceae*. Its growth is possible both photoheterotrophically under anoxic conditions and chemotrophically under oxic conditions in the dark or under anoxic conditions by denitrification (1)*. Rhodoplanes* sp. strain T2.26MG-98 was isolated from a strictly anaerobic methanogenic enrichment culture using a 492.6-m-deep core sample obtained from a drilling project designed to identify the microbially diverse populations existing in the deep subsurface of the Iberian Pyrite Belt (southwestern Spain) (2). The borehole coordinates were 37^°^43′45.42″N, 6^°^33′23.57″W. Drilling was performed as described in reference 3. A powdered rock sample (∼5 g) was used as inoculum. The isolation and growth of *Rhodoplanes* sp. strain T2.26MG-98 were done under the conditions described in reference 4.

Genomic DNA of strain T2.26MG-98 was extracted and the 16S rRNA gene amplified under the conditions described in reference 5. Reads were edited and assembled by Mega X (6) and Bioedit 7.0.4 (7). The complete 16S rRNA gene sequence was compared with sequences on other GenBank databases on NCBI using BLAST (8). The closest sequence was found to belong to Rhodoplanes piscinae JA266 (98.96%), a facultative anaerobic microorganism (9).

Genomic DNA was sequenced using the Illumina MiSeq platform, with 39.6× mean coverage. The sequencing run generated 533,028 2 × 250-paired-end reads with a mean length of 503 bp. The reads were trimmed by Trimmomatic 0.36, and quality analyses were performed for the reads using FastQC v.0.11.8 software (http://www.bioinformatics.babraham.ac.uk/projects/fastqc/). *De novo* assembly was carried out using the software SPAdes 3.9.0 (10), and the assembly of extrachromosomal genetic elements was carried out using Recycler (11). Plasmid contigs were aligned against the chromosomal assembly with Mauve Aligner 2.4.0 (12). Contigs were ordered and new scaffolds were created by means of SSPACE software (13) using the genome of Rhodoplanes piscinae DSM 19946 as the reference (GenBank accession number NPEW00000000). This yielded a chromosome in 214 scaffolds, with an *N*_50_ of 50.61 kb, a GC content of 70.24%, one chromosome of 5,589,602 bp, and one plasmid of 53,767 bp in a single contig. Default parameters were used for all software unless otherwise specified.

Gene prediction and annotation were carried out using three methods, PROKKA v1.12 software (14), BlastKOALA v2.1 (15), and the RAST platform (16), using Rhodoplanes piscinae DSM 19946 as a reference genome. The CRISPRCasFinder Web server was used to find clustered regularly interspaced short palindromic repeat (CRISPR) structures (17). A total of 4,994 coding DNA sequences, 57 tRNA genes, 1 rRNA operon, 1 transfer-messenger RNA (tmRNA), 445 signal peptides, and 4 CRISPR sequences were identified. A complete set of genes encoding nitrate ammonification, denitrification, and sulfur oxidation, as well as thiosulfate reduction, was detected. A gene encoding a periplasmic iron oxidase (UniProtKB accession number P50500) identified in Acidithiobacillus ferrooxidans (18) was also detected in the genome. The strain also presents genes encoding the reduction of perchlorate, an activity of astrobiological interest (19). Regarding annotation of the 53.7-kb plasmid, 69 coding genes were identified. Genes involved in the regulation of the plasmid copy number (*repA*), one signal peptide, and one tRNA were identified.

The analysis of the genome of *Rhodoplanes* sp. strain T2.26MG-98 should be helpful in identification of the mechanisms used for life to develop in the extreme oligotrophic conditions existing in the dark biosphere.

### Data availability.

Reads have been deposited at DDBJ/ENA/GenBank under the accession number ERR2843940, and the complete genome sequences and annotations have been deposited under the accession numbers UWOC00000000 for this chromosome, LR026982 for this plasmid, and GCF_900604295 for the annotation. The version described in this paper is the first version.
